# Vertical tank capacity measurement based on Monte Carlo method

**DOI:** 10.1371/journal.pone.0250207

**Published:** 2021-04-16

**Authors:** Guoyu Chen, Yong Wan, Huanhuan Lin, Houlin Hu, Guixiong Liu, Yanhua Peng

**Affiliations:** 1 Research and Development Department, Guangzhou Institute of Energy Testing, Guangzhou, China; 2 School of Mechanical & Automotive Engineering, South China University of Technology, Guangzhou, China; 3 School of Mechanical and Electrical Engineering, Guilin University of Electronic Technology, Guilin, China; University of Glasgow, UNITED KINGDOM

## Abstract

Vertical tanks are commonly used appliances for liquids, and its capacity is very important for quantitative liquid ratio and liquid trade. In order to measure the capacity of vertical tanks more conveniently, this paper proposes a vertical tank capacity measurement method based on Monte Carlo Method. The method arranges a plurality of sensor points on the inner surface of the tank, and then performs Monte Carlo tests by generating a large number of random sample points, and finally calculates the capacity by counting the sample points that meet the criterion. The criterion for whether a sample point is located in the tank, which is the core issue, is established with the coordinates of sensor points and the distance between different sensor points along the surface of the tank. The results show that the absolute error of the measurement results of the proposed method does not exceed ±0.0003[m^3^], and the absolute error of capacity per unit volume has a linear relationship with the height of the vertical tank, and has little effect with the radial size of the vertical tank.

## Introduction

Vertical tank is widely used in the storage, transportation and measurement of liquid substances such as petroleum and chemical materials. Accurate measurement of the vertical tank capacity is directly related to the fairness of liquid substance transactions.

One of the commonly methods used to measure the capacity of a vertical tank is Geometric Measurement Method (GMM). GMM uses steel tape, theodolite or other measuring device to measure tank’s geometry, and use dedicated computer software to process the measured data to obtain the tank capacity [1–3]. This method is with a low level of automation, requires measuring a range of geometric parameters, such as the height, circumference, and diameter of different rings (vertical tanks are actually composed of many rings), which is of a heavy work load and time-consuming. Due to the need for a lot of manual operations, the measurement efficiency of this method is low, and the measurement results are easily affected by operator errors.

Another commonly used method to measure the capacity of a vertical tank is Volumetric Method (VM). VM compares the capacity difference between a tank with higher precision and the vertical tank being measured [1, 4]. During measurement, a fixed volume of water is poured into the storage tank through a standard metal tank (apparatus with a higher level of accuracy), and then the capacity table is calculated by interpolation. This method can be adapted to irregularly shaped tanks, and the capacity can be obtained directly through volume comparison without conversion calculation. VM is with high precision and simple to operate, but requires liquid (usually is water) supply while measurement and the contaminated liquid needs further processing while measurement finish. At present, 5m^3^ is already a very large standard metal tank, but the volume range of vertical tanks includes 20m^3^~700m^3^, even more than 700m^3^, and the height can exceeds 30 meters. To place the standard metal tank higher than the metal tank, this is difficult to achieve. In addition, the standard metal tank is too small compared to the vertical tank, and it takes a lot of time to pour a fixed amount of water many times. If the vertical tank is large, VM is rarely used because it takes a long time and consumes large amounts of water.

With the development of technology, new techniques for measuring vertical tank capacity has emerged. Laser Scanner Method (LSM) reconstructs the internal volume of a vertical tank by emitting laser light to the surroundings [5–7]. The principle of LSM is similar to that of GMM. They both obtain the vertical tank capacity by measuring the geometry parameters, but LSM is more automated. In LSM, a laser scanner is used instead of the manual measurement, and the geometric dimensions of the vertical tank are get by fitting the points cloud obtained through multiple scans. LSM includes external scanning and internal scanning. External scanning is to place the laser scanner outside the vertical tank, which requires an open field of view and as few obstructions as possible. Internal scanning is to place the laser scanner in a vertical tank, which requires fewer parts present inside the vertical tank, and the bottom of the vertical tank keeps stable during the measurement. However, when the vertical tank is large, the inspector stepping on the bottom plate may cause the bottom of the vertical tank to undulate and deform, causing the laser scanner to be unstable.

The volumetric method, geometric measurement method, and laser scanner method are used to measure vertical tanks, with a relatively fixed cycle, about once every four years. If you want to monitor the capacity of the vertical tank in real time, it is a more feasible way to arrange sensors in the vertical tank.

Monte Carlo Method (MCM) constructs a probabilistic model that approximates the performance of the system and performs random experiments on a digital computer [8]. It is very common to calculate the solid volume represented by the boundary with MCM [9, 10]. Currently when using MCM to measure a volume, the boundary is usually obtained by laser scanning, which can provide the boundary points cloud. And with octrees construction on the points cloud, whether a point is located in the model is classified [11]. How to construct the boundary conditions of Monte Carlo experiment is the core problem of this method. MCM is computationally intensive, and still need environmental stability while laser scanning. If the boundary discrimination condition of MCM can be simplified, the amount of calculation would be greatly reduced. As General Conference of Weights & Measures has redefined SI base units and associated the definition of these basic units with physical constants, measurement standards may be integrated into the chip. That is to say, it is possible to describe the boundary discrimination condition of MCM through inserting the chip into the vertical tank. In the future, vertical tank capacity measurement would develop towards intelligent, real-time online monitoring. Under this background, we propose a vertical tank capacity measurement method based on the Monte Carlo method, hoping to conveniently measure the vertical tank capacity and obtain acceptable results, and do preliminary theoretical research for the future real-time monitoring of the change in vertical tank capacity. This paper is an exploratory study on the mathematical model and experiment of measuring vertical tank capacity by Monte Carlo method. This method focuses on constructing vertical tank boundary and capacity measurements on the premise of knowing the coordinates of certain points on the inner surface of the vertical tank.

## Methods

**Fig 1:**
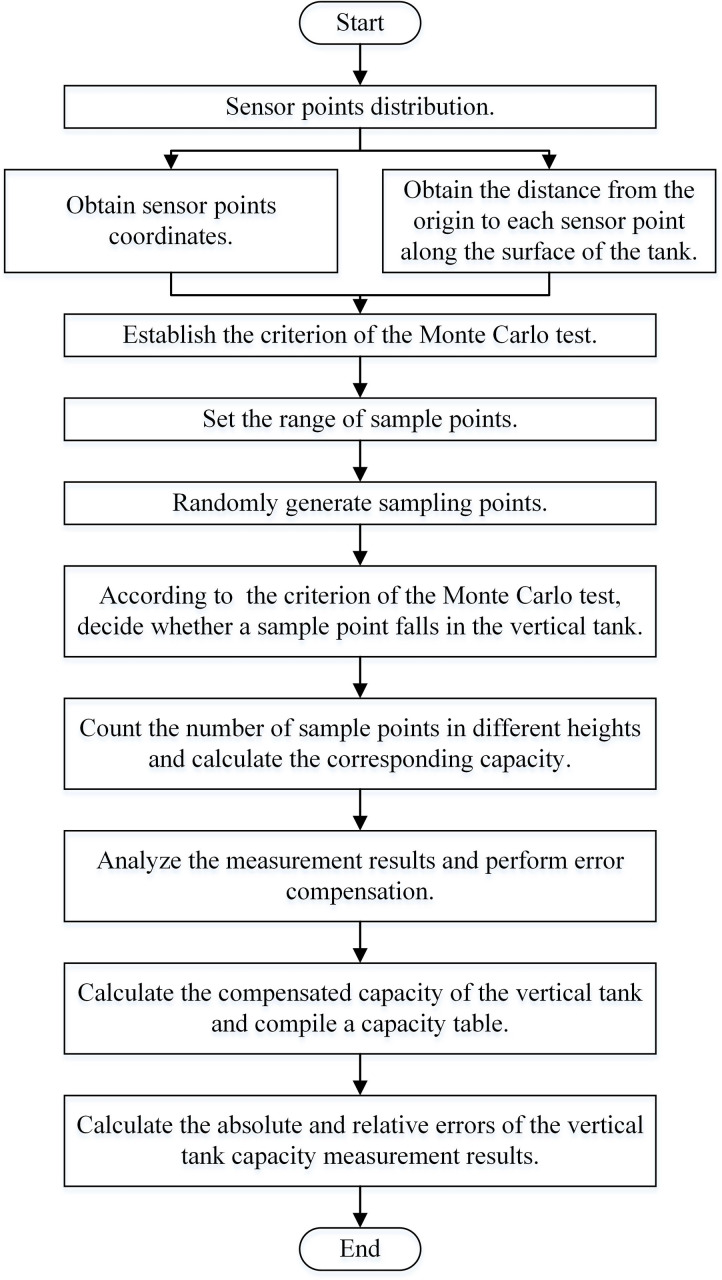
Flow chart of the proposed method.

Vertical tank capacity measurement based on Monte Carlo method is to arrange sensor points on the inner surface of the vertical tank. And then, according to the coordinates of the sensor points and the distance between each point along the tank surface, criterion to decide whether a sample point is located in the vertical tank is established. Base on this criterion, the number of sample points falling in different heights is counted, and the capacity value represented by different liquid level is calculated with the number of sample points. Fig 1 shows the main steps of the proposed method.

### The distribution of sensor points

**Fig 2:**
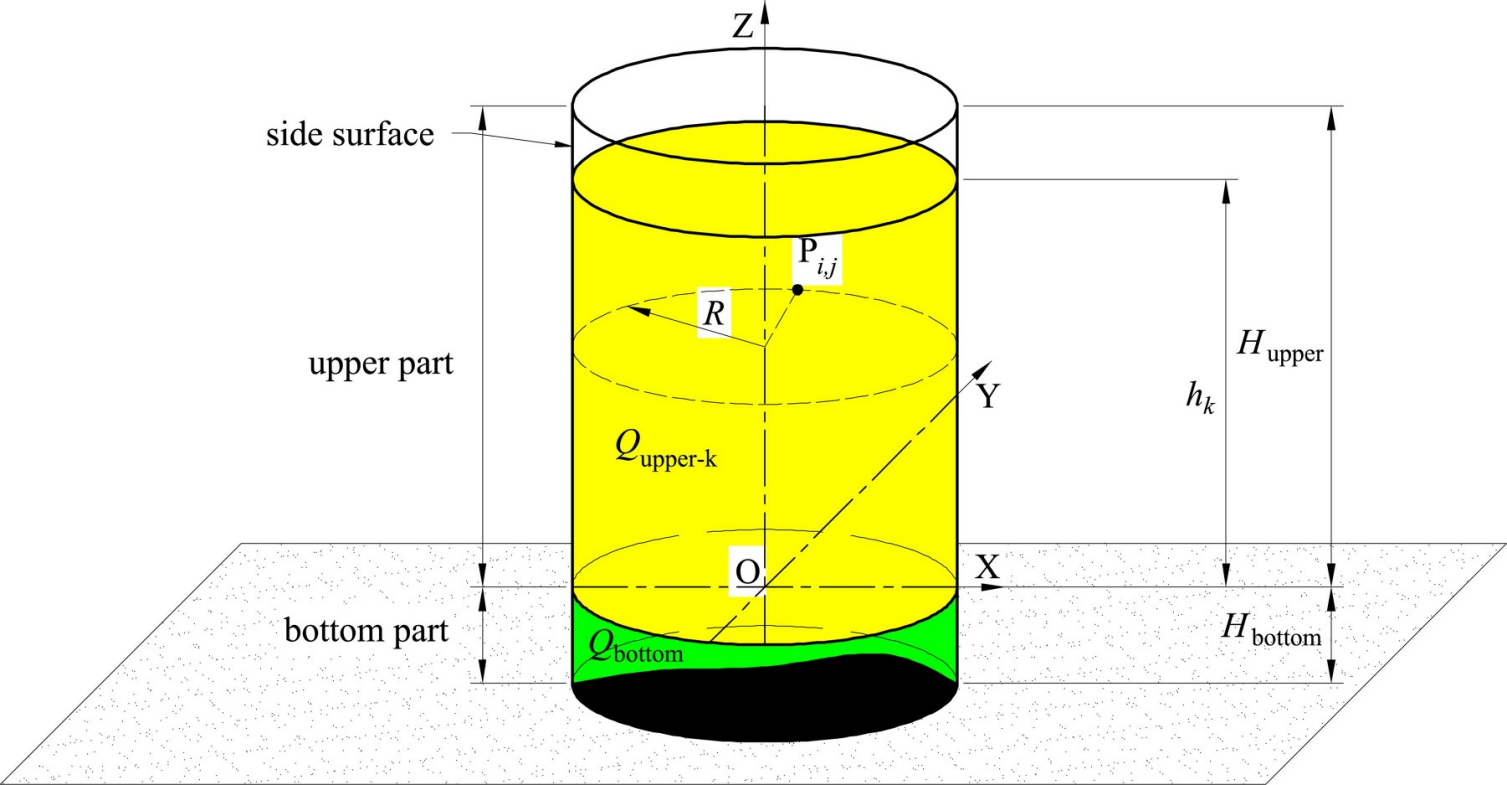
The coordinate system.

**Fig 2** is the coordinate system. Vertical tanks are generally cylindrical, and their cross-sections are generally circular. Therefore, our methods and tests are all carried out with circular cross-sections. Vertical tank is divided into upper part and bottom part. The bottom part of vertical tank is usually irregular because of ground subsidence or other reasons, and the capacity of bottom part is obtained by volumetric method or other methods. The bottom of the vertical tank generally only occupies little share of the entire storage tank, and the bottom volume is only used when the liquid is first fed or when the tank is cleared. In the daily custody transfer, the capacity table on the upper part of the vertical tank is generally used for calculation, and the amount of liquid in each custody transfer is generally not less than two meters in the height of the vertical tank. Due to the deformation of the bottom plate and other reasons, it is difficult to measure the bottom volume of the vertical tank very accurately by the geometric measurement method and the laser scanner method, and more attention is paid to the measurement of the upper part of the storage tank. Thus, this paper focuses on acquiring the capacity of upper part. The height of bottom part is *H*_bottom_, and the height of upper part is *H*_upper_. The inner radius of the vertical tank is *R*. The capacity of the vertical tank is synthesized from the upper part and the bottom part, shown in Eq 1.

$$Q_{k}=Q_{\text{upper-}k}+Q_{\text{bottom}}.$$ (1)

where *Q*_*k*_ is the vertical tank capacity when the liquid level is *h*_*k*_. *Q*_upper-*k*_ is the capacity of upper part when the liquid level is *h*_*k*_. *Q*_bottom_ is the capacity of bottom part.

**Fig 3:**
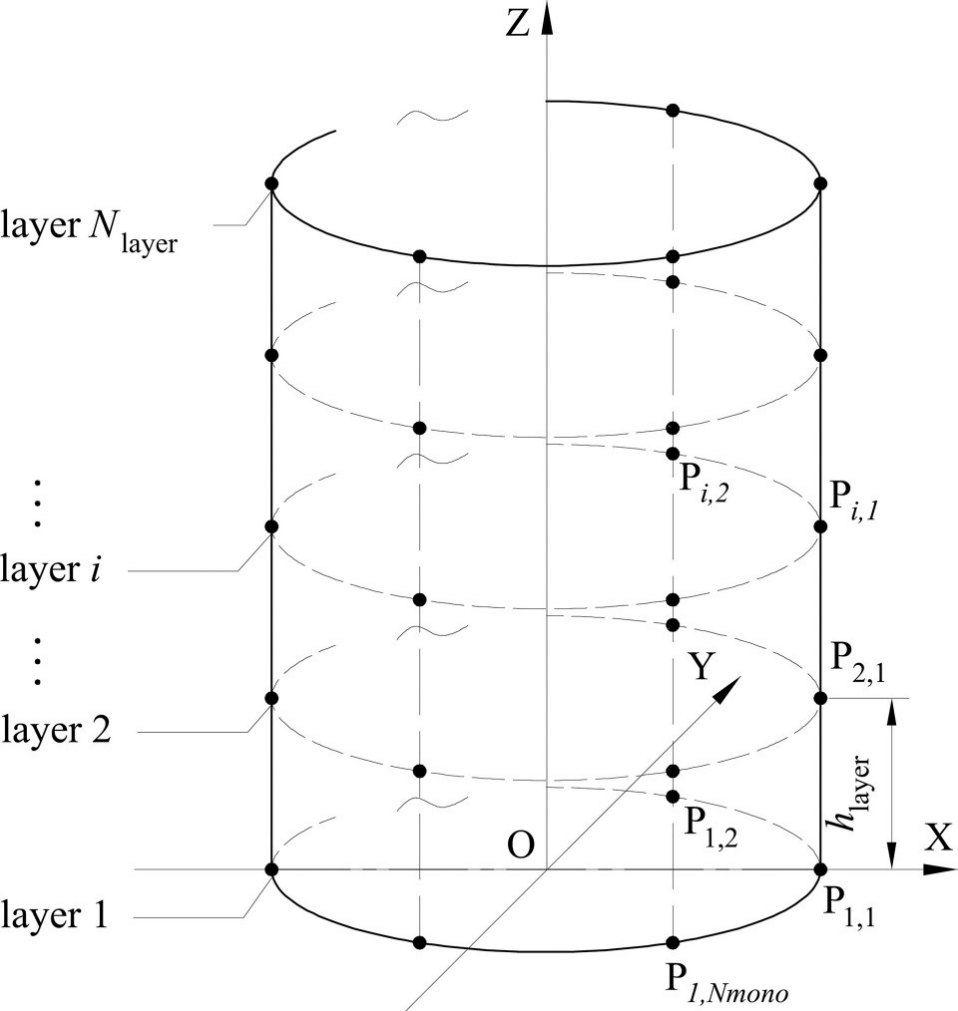
Sensor points distribution.

**Fig 3** shows the distribution of sensor points. Sensor points are equiangularly arranged on different layers. The number of layers is *N*_layer_ and the number of sensor points on each layer is *N*_mono_. The projection of the sensor points of each layer on O-XY surface are coincidence. The interval between layers is *h*_layer_ and the total number of sensor points is *N*_sensor_. Thus,

$$h_{\text{layer}}=H_{\text{upper}}/\left(N_{\text{layer}}-1\right).$$ (2)

$$N_{\text{sensor}}=N_{\text{mono}}\cdot N_{\text{layer}}.$$ (3)

The sensor points in *Layer*−*i* are numbered as P_*i*,1_, P_*i*,2_,…, ${\text{P}}_{i,N_{\text{mono}}}$in the counter clockwise direction, where 1≤ *i* ≤ *N*_layer_. The coordinates of sensor point P_*i*,*j*_ are (*x*_*i*,*j*_, *y*_*i*,*j*_, *z*_*i*,*j*_), where 1≤ *i* ≤ *N*_layer_, 1≤ *j* ≤ *N*_mono_. Specially, *Layer*−1 and *Layer*−*N*_layer_ are the lowest and highest positions of upper part respectively, and *z*_1,*j*_ = 0, $z_{N_{\text{layer}},j}=H_{\text{upper}}$.

### Criterion of the Monte Carlo test

**Fig 4:**
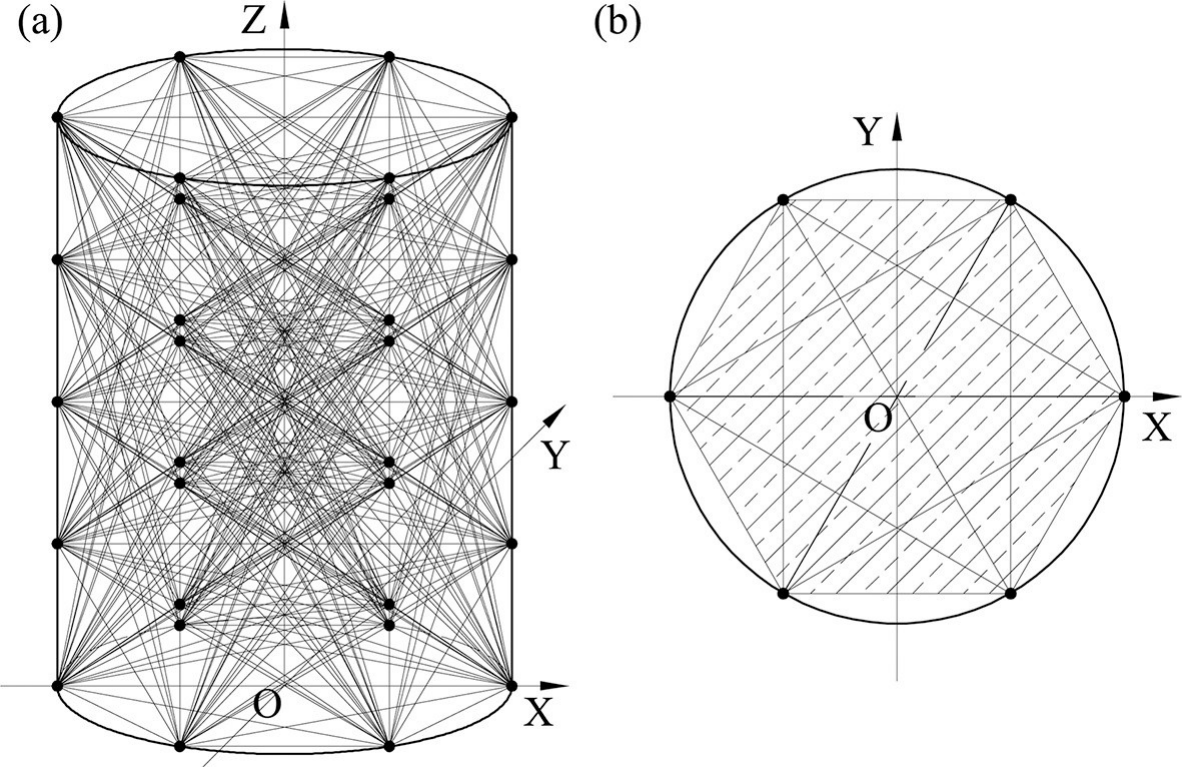
The enclosed area formed by all sensor points. (a) 3D illustration of the enclosed space; (b) The projection of the enclosed space on O-XY surface.

Criterion of the Monte Carlo test is used to decide whether a sample point falls in the vertical tank. As the vertical tank is convex and the sensor points are located on the inner surface of the tank, sample points in the enclosed area formed by all sensor points must be in the tank, shown in **Fig 4**. If sample point P_sample_ locates in the enclosed area formed by all sensor points, we have *a*, *b* and *c*, make

$$\vec{{\text{OP}}_{\text{sample}}}=a\cdot \vec{{\text{OP}}_{i1,j1}}+b\cdot \vec{{\text{OP}}_{i2,j2}}+c\cdot \vec{{\text{OP}}_{i3,j3}}.$$ (4)

where *a*+*b*+*c*≤1, 0≤*a*≤1, 0≤*b*≤1, 0≤*c*≤1. P_*i*1,*j*1_,P_*i*2,*j*2_andP_*i*3,*j*3_ are sensor points, 1≤ *i*1 ≤ *N*_layer_, 1≤ *j*1 ≤ *N*_mono_, 1≤ *i*2 ≤ *N*_layer_, 1≤ *j*2 ≤ *N*_mono_, $\vec{{\text{OP}}_{i1,j1}}\neq \vec{{\text{OP}}_{i2,j2}}\neq \vec{{\text{OP}}_{i3,j3}}$.

If we cannot find *a*, *b* and *c* let P_sample_ satisfies Eq 4, it is still uncertain whether P_sample_ falls in the vertical tank or not. The subsequent calculation will be performed.

**Fig 5:**
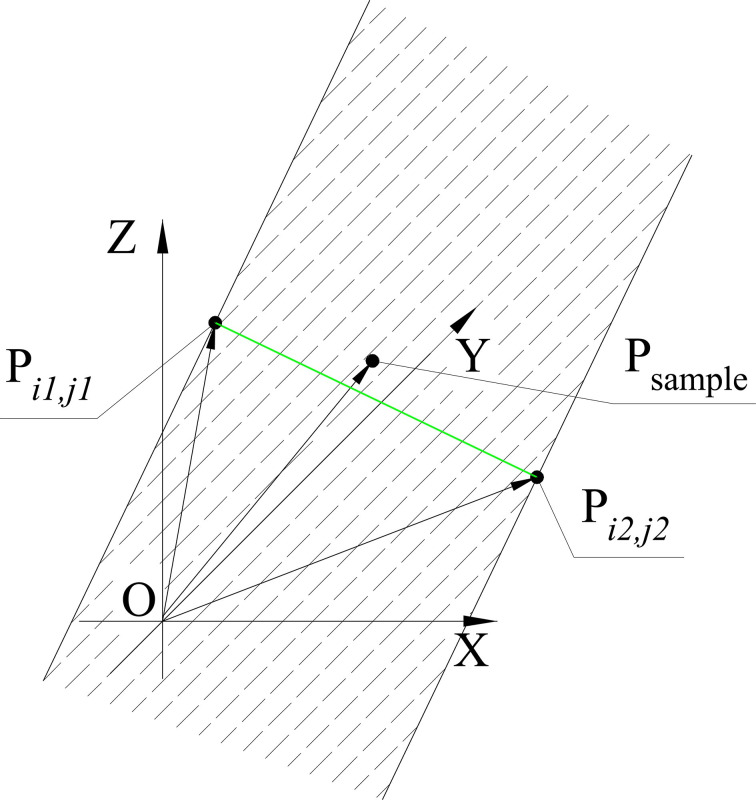
Positional relationship of P_sample_, P_*i*1,*j*1_, and P_*i*2,*j*2_.

**Fig 5** is the positional relationship of P_sample_,P_*i*1,*j*1_, and P_*i*2,*j*2_. If P_sample_ is between P_*i*1,*j*1_, and P_*i*2,*j*2_(i.e. P_sample_ falls within the shaded area in Fig 4), we have

$$\vec{{\text{P}}_{i1,j1}{\text{P}}_{\text{sample}}}\cdot \vec{{\text{P}}_{i1,j1}{\text{P}}_{i2,j2}}\geq 0.$$ (5)

$$\vec{{\text{P}}_{i2,j2}{\text{P}}_{\text{sample}}}\cdot \vec{{\text{P}}_{i2,j2}{\text{P}}_{i1,j1}}\geq 0.$$ (6)

The distance from P_sample_ to Line P_*i*1,*j*1_ P_*i*2,*j*2_ is $\vec{{\text{P}}_{i1,j1}{\text{P}}_{\text{sample}}}\cdot \vec{{\text{P}}_{i1,j1}{\text{P}}_{i2,j2}}/\left\|\vec{{\text{P}}_{i1,j1}{\text{P}}_{i2,j2}}\right\|$, where $\left\|\vec{{\text{P}}_{i1,j1}{\text{P}}_{i2,j2}}\right\|$is the modulus of $\vec{{\text{P}}_{i1,j1}{\text{P}}_{i2,j2}}$. Find P_*i*1,*j*1_ and P_*i*2,*j*2_ makes $\vec{{\text{P}}_{i1,j1}{\text{P}}_{\text{sample}}}\cdot \vec{{\text{P}}_{i1,j1}{\text{P}}_{i2,j2}}/\left\|\vec{{\text{P}}_{i1,j1}{\text{P}}_{i2,j2}}\right\|$ minimal, that is

$$distance=\min\left\{\frac{\vec{{\text{P}}_{i1,j1}{\text{P}}_{\text{sample}}}\cdot \vec{{\text{P}}_{i1,j1}{\text{P}}_{i2,j2}}}{\left\|\vec{{\text{P}}_{i1,j1}{\text{P}}_{i2,j2}}\right\|}\right\}.$$ (7)

where 1≤ *i*1 ≤ *N*_layer_, 1≤ *j*1 ≤ *N*_mono_, 1≤ *i*2 ≤ *N*_layer_, 1≤ *j*2 ≤ *N*_mono_, $\vec{{\text{OP}}_{i1,j1}}\neq \vec{{\text{OP}}_{i2,j2}}$.

**Fig 6:**
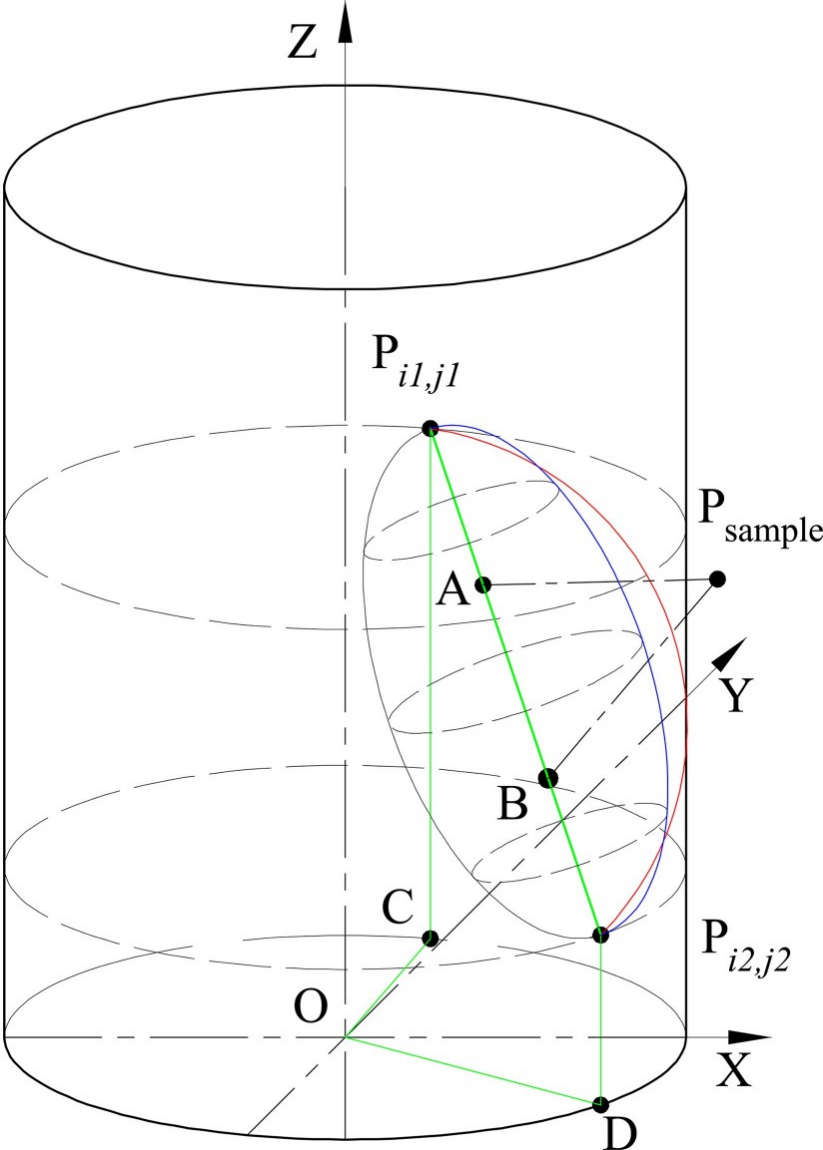
The ellipsoid with P_i1,j1_, and P_i2,j2_ as the endpoints.

After finding out two sensor points making *distance* is the smallest, for example, P_*i*1,*j*1_ and P_*i*2,*j*2_ satisfy Eqs 5, 6, and 7, we construct an ellipsoid with P_*i*1,*j*1_ and P_*i*2,*j*2_ as the endpoints, shown in **Fig 6**. Point A and B are the foci of the ellipse. Thus, $\vec{{\text{P}}_{i1,j1}\text{A}}=\lambda \cdot \vec{{\text{P}}_{i1,j1}{\text{P}}_{i2,j2}}$, $\vec{{\text{P}}_{i1,j1}\text{B}}=\left(1-\lambda \right)\cdot \vec{{\text{P}}_{i1,j1}{\text{P}}_{i2,j2}}$, where *λ* is a scale factor, 0 < *λ* < 1.

As $\vec{\text{OA}}=\vec{{\text{OP}}_{i1,j1}}+\vec{{\text{P}}_{i1,j1}\text{A}}=\vec{{\text{OP}}_{i1,j1}}+\lambda \cdot \vec{{\text{P}}_{i1,j1}{\text{P}}_{i2,j2}}$ and $\vec{\text{OB}}=\vec{{\text{OP}}_{i1,j1}}+\vec{{\text{P}}_{i1,j1}\text{B}}=\vec{{\text{OP}}_{i1,j1}}+\left(1-\lambda \right)\cdot \vec{{\text{P}}_{i1,j1}{\text{P}}_{i2,j2}}$, we have the coordinates of Point A are *x*_A_ = *x*_*i*1,*j*1_+*λ* · (*x*_*i*2,*j*2_ –*x*_*i*1,*j*1_), *y*_A_ = *y*_*i*1,*j*1_+*λ* · (*y*_*i*2,*j*2_ –*y*_*i*1,*j*1_), *z*_A_ = *z*_*i*1,*j*1_+*λ* · (*z*_*i*2,*j*2_ –*z*_*i*1,*j*1_). The coordinates of Point B are *x*_B_ = *x*_*i*1,*j*1_+(1-*λ*) · (*x*_*i*2,*j*2_ –*x*_*i*1,*j*1_), *y*_B_ = *y*_*i*1,*j*1_+(1-*λ*) · (*y*_*i*2,*j*2_ –*y*_*i*1,*j*1_), *z*_B_ = *z*_*i*1,*j*1_+(1-*λ*) · (*z*_*i*2,*j*2_ –*z*_*i*1,*j*1_). Then the ellipsoid equation is

$$\begin{array}{l} \sqrt{{\left(x-x_{\text{A}}\right)}^{2}+{\left(y-y_{\text{A}}\right)}^{2}+{\left(z-z_{\text{A}}\right)}^{2}}+\\ \sqrt{{\left(x-x_{\text{B}}\right)}^{2}+{\left(y-y_{\text{B}}\right)}^{2}+{\left(z-z_{\text{B}}\right)}^{2}}=\left\|\vec{P_{i1,j1}P_{i2,j2}}\right\|. \end{array}$$ (8)

Once *λ* is solved out, Eq 8 would be unique. Following will solve the *λ* value.

In Fig 6, the distance from O to P_*i*1,*j*1_ and P_*i*2,*j*2_ along the surface of the tank are $l_{i1,j1}=\left\|\vec{\text{OC}}\right\|+\left\|\vec{{\text{CP}}_{i1,j1}}\right\|$ and $l_{i2,j2}=\left\|\vec{\text{OD}}\right\|+\left\|\vec{{\text{DP}}_{i2,j2}}\right\|$. The distance from P_*i*1,*j*1_ to P_*i*2,*j*2_ along the surface of the tank is defined as *l*_*i*1,*j*1↔*i*2,*j*2_. Since the surface continuity of the vertical tank, approximate solution to get *l*_*i*1,*j*1↔*i*2,*j*2_ is

$$l_{i1,j1↔i2,j2}=\left\|\vec{{\text{P}}_{i1,j1}{\text{P}}_{i2,j2}}\right\|\cdot \left(\frac{l_{i1,j1}/\left\|\vec{{\text{OP}}_{i1,j1}}\right\|+l_{i2,j2}/\left\|\vec{{\text{OP}}_{i2,j2}}\right\|}{2}\right).$$ (9)

According to [12], the half circumference of the ellipsoid is

$$C_{\text{half}}=\frac{8\pi \cdot {\left(L_{\text{major}}+L_{\min\text{or}}\right)}^{4}-0.375\pi \cdot {\left(L_{\text{major}}-L_{\min\text{or}}\right)}^{4}}{8\left(L_{\text{major}}+L_{\min\text{or}}\right)\cdot \left(4{\left(L_{\text{major}}+L_{\min\text{or}}\right)}^{2}-{\left(L_{\text{major}}-L_{\min\text{or}}\right)}^{2}\right)}.$$ (10)

where *L*_major_ is the ellipsoid major axis length, *L*_minor_ is the ellipsoid minor axis length. $L_{\text{major}}=\left\|\vec{{\text{P}}_{i1,j1}{\text{P}}_{i2,j2}}\right\|$, $L_{\text{minor}}=\sqrt{\lambda -\lambda^{2}}\cdot L_{\text{major}}=\sqrt{\lambda -\lambda^{2}}\cdot \left\|\vec{{\text{P}}_{i1,j1}{\text{P}}_{i2,j2}}\right\|$.

Solving *C*_half_ = *l*_*i*1,*j*1↔*i*2,*j*2_, we can get *λ* and the ellipsoid equation, i.e. Eq 8.

According to the definition of an ellipsoid, if the sum of the distance from P_sample_ to A and B is shorter than *L*_minor_, P_sample_ is in the ellipsoid. That is, if

$$\left\|\vec{{\text{P}}_{\text{sample}}\text{A}}\right\|+\left\|\vec{{\text{P}}_{\text{sample}}\text{B}}\right\|\leq \left\|\vec{{\text{P}}_{i1,j1}{\text{P}}_{i2,j2}}\right\|,$$ (11)

we think that P_sample_ falls in the vertical tank.

In summary, if a sample point P_sample_ falls in the vertical tank, we have

1. The coordinates of P_sample_ satisfy Eq 1.
2. If the coordinates of P_sample_ do not satisfy Eq 1, they should satisfy Eq 11.

### The vertical tank capacity

When conducting the Monte Carlo tests, sample points are randomly generated in the smallest cuboid that contains the vertical tank. If *x*_min_ = min{*x*_*i*,*j*_}, *y*_min_ = min{*y*_*i*,*j*_}, *z*_min_ = min{*z*_*i*,*j*_}, *x*_max_ = max{*x*_*i*,*j*_}, *y*_max_ = max{*y*_*i*,*j*_}, *z*_max_ = max{*z*_*i*,*j*_}, where 1≤ *i* ≤ *N*_layer_, 1≤ *j* ≤ *N*_mono_, *x*_*i*,*j*_, *y*_*i*,*j*_ and *z*_*i*,*j*_ are the coordinates of sensor points, the coordinates of sample points would be *x*_min_≤*x*≤*x*_max_, *y*_min_≤*y*≤*y*_max_, *z*_min_≤*z*≤*z*_max_.

After the Monte Carlo tests, if the number of sample points satisfying the criterions of the Monte Carlo Test is *N*_IN_, and totally *N*_sample_ sample points are generated, the capacity of the upper part would be

$$Q_{\text{upper}}=\frac{N_{\text{IN}}}{N_{\text{sample}}}\cdot \left(x_{\max}-x_{\min}\right)\cdot \left(y_{\max}-y_{\min}\right)\cdot \left(z_{\max}-z_{\min}\right).$$ (12)

Similarly, for a certain height *h*_*k*_, if the number of the sample points that satisfy the criterions of the Monte Carlo Test and *z* ≤ *h*_*k*_ is *N*_IN-*k*_, the capacity in upper part at *h*_*k*_ is

$$Q_{\text{upper-}k}=\frac{N_{\text{IN-}k}}{N_{\text{sample}}}\cdot \left(x_{\max}-x_{\min}\right)\cdot \left(y_{\max}-y_{\min}\right)\cdot \left(z_{\max}-z_{\min}\right).$$ (13)

The vertical tank capacity at *h*_*k*_ is

$$Q_{k}=Q_{\text{bottom}}+Q_{\text{upper-}k}.$$ (14)

The tank capacity table can be compiled by counting the number of sample points satisfying the criterions of the Monte Carlo Test at different heights.

### The Monte Carlo test

**Fig 7:**
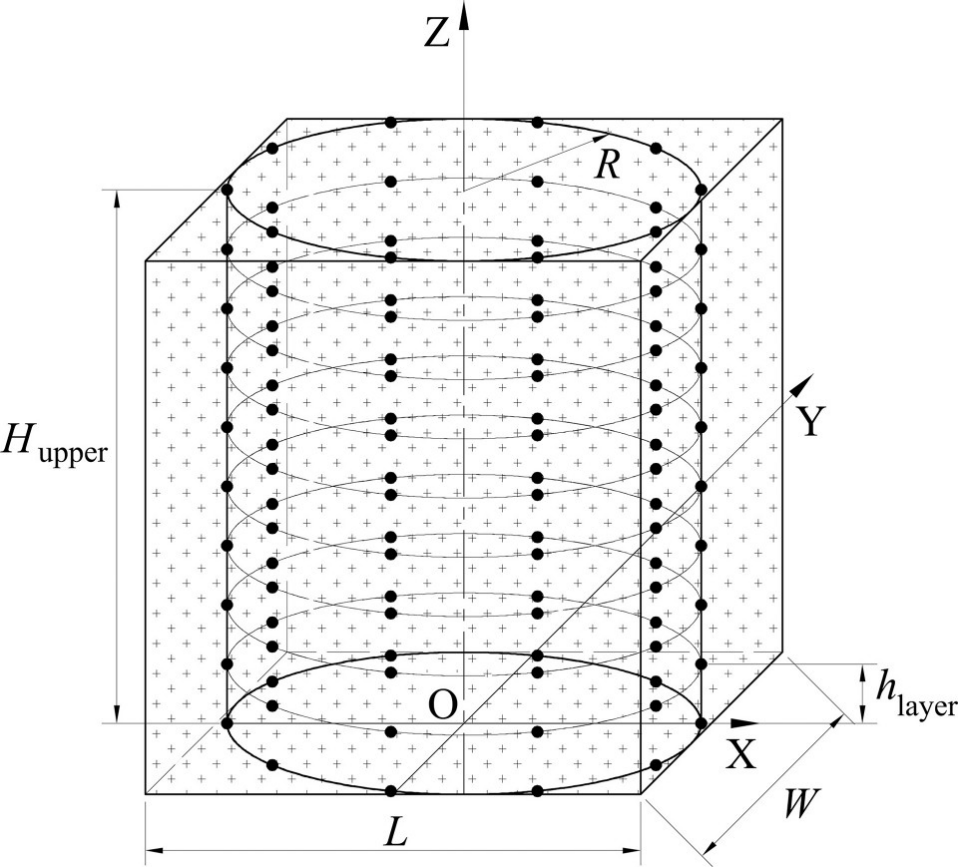
The vertical tank model.

**Fig 7** is the vertical tank model of the Monte Carlo test. The radius of vertical tank is *R* = 1.300[m], and the upper part height of the vertical tank is *H*_upper_ = 8.476[m]. The maximum capacity of upper part is π · *R*^2^ · *H*_upper_ ≈ 45.000[m^3^]. Sensor points are distributed in ten layers (*N*_layer_ = 10). Each layer has ten sensor points uniformly distributed equiangularly (*N*_mono_ = 10). Thus, *N*_sensor_ = *N*_mono_ · *N*_layer_ = 100, i.e., there are 100 sensor points in total. Considering the size of the vertical tank, we initially set up 100 sensor points. The influence of the number of sensor points on the measurement results will be further studied in the follow-up work. Currently, 100 sensor points are used to explore the feasibility of the proposed method. The difference of heights between each layer is *h*_layer_ = 0.8476[m]. Sample points are generated in Cuboid *L*–*W*–*H*_upper_, whose *L* = 2.600[m], *W* = 2.600[m] and *H*_upper_ = 8.476[m]. Thus, *x*_min_ = -1.300[m], *y*_min_ = -1.300[m], *z*_min_ = 0, *x*_max_ = 1.300[m], *y*_max_ = 1.300[m], *z*_max_ = 8.4776[m], and the coordinates of sample points satisfy -1.300[m]≤*x*≤1.300[m], -1.300[m]≤*y*≤1.300[m], 0≤*z*≤8.476[m].

The Monte Carlo tests are performed on Matlab, and the sample points are generated with Matlab’s built-in random function ─ *unifrnd ()*. Totally *N*_sample_ = 10^6^ sample points are generated in each test. A total of 10 tests were conducted.

In order to discuss the measurement results of vertical tanks of different volumes, the absolute error of capacity per unit volume is calculated as

$$\varepsilon_{k}=\left(Q_{k}-{Q^{\prime }}_{k}\right)/V_{\text{cuboid}},$$ (15)

where *Q*_*k*_ is the vertical tank capacity at *h*_*k*_. ${Q^{\prime }}_{k}$is the actual capacity of vertical tank at *h*_*k*_, ${Q^{\prime }}_{k}=\pi \cdot R^{2}\cdot h_{k}+Q_{\text{bottom}}$. *V*_cuboid_ is the volume of cuboid, *V*_cuboid_ = *L* · *W* · *H*_upper_.

**Fig 8:**
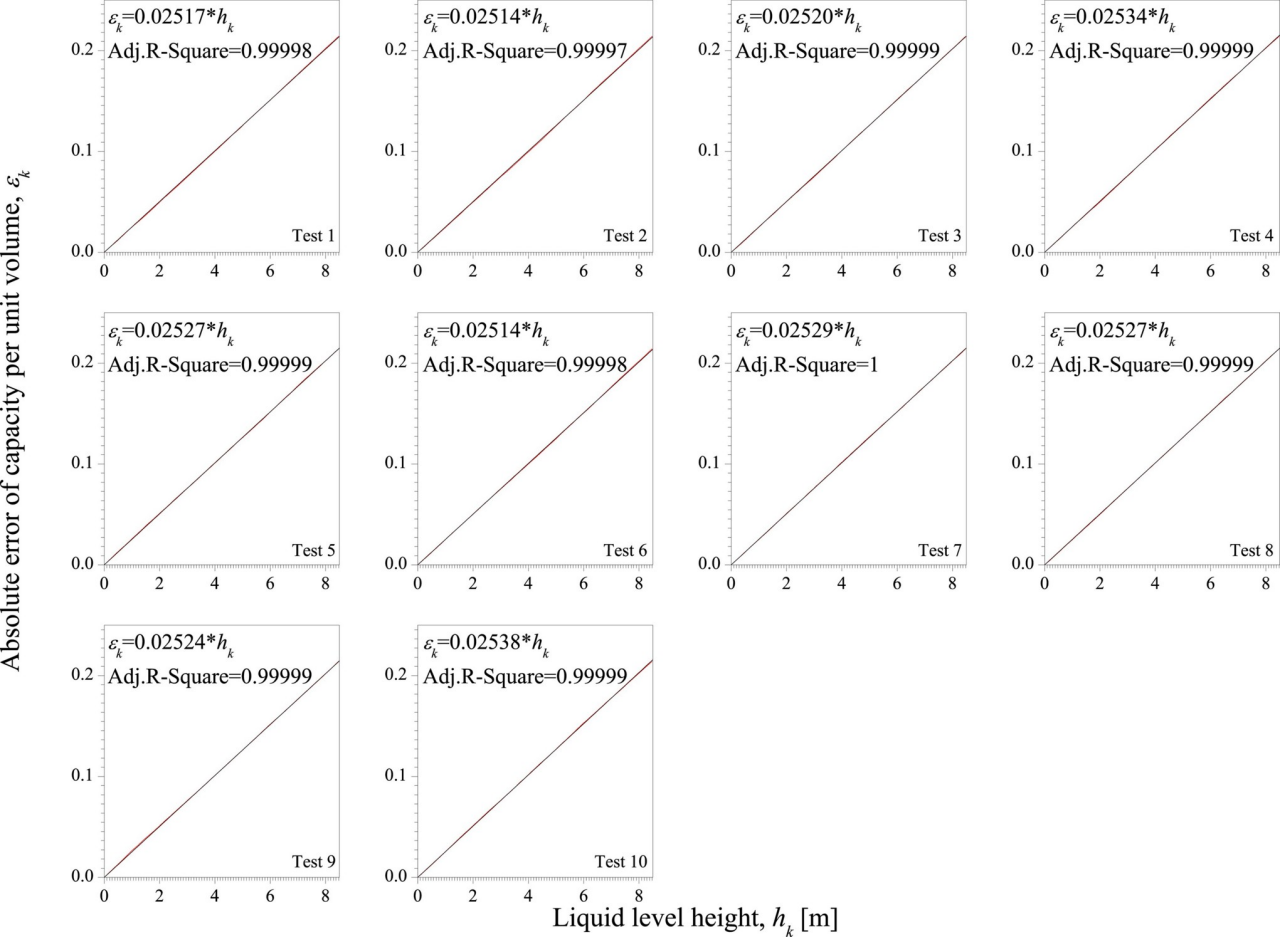
The absolute error of capacity per unit volume of Test 1~10.

**Fig 8** is the absolute error of capacity per unit volume of Test 1~10. It shows that there is a significant linear relationship between *ε*_*k*_ and *h*_*k*_ in each test. The slopes of the fitting results of Test 1~10 are all around 0.0252.

To further investigate the relationship between the absolute error of capacity per unit volume and the size of the vertical tank, another sixty tests are carried out for different heights and radii of tank.

**Fig 9:**
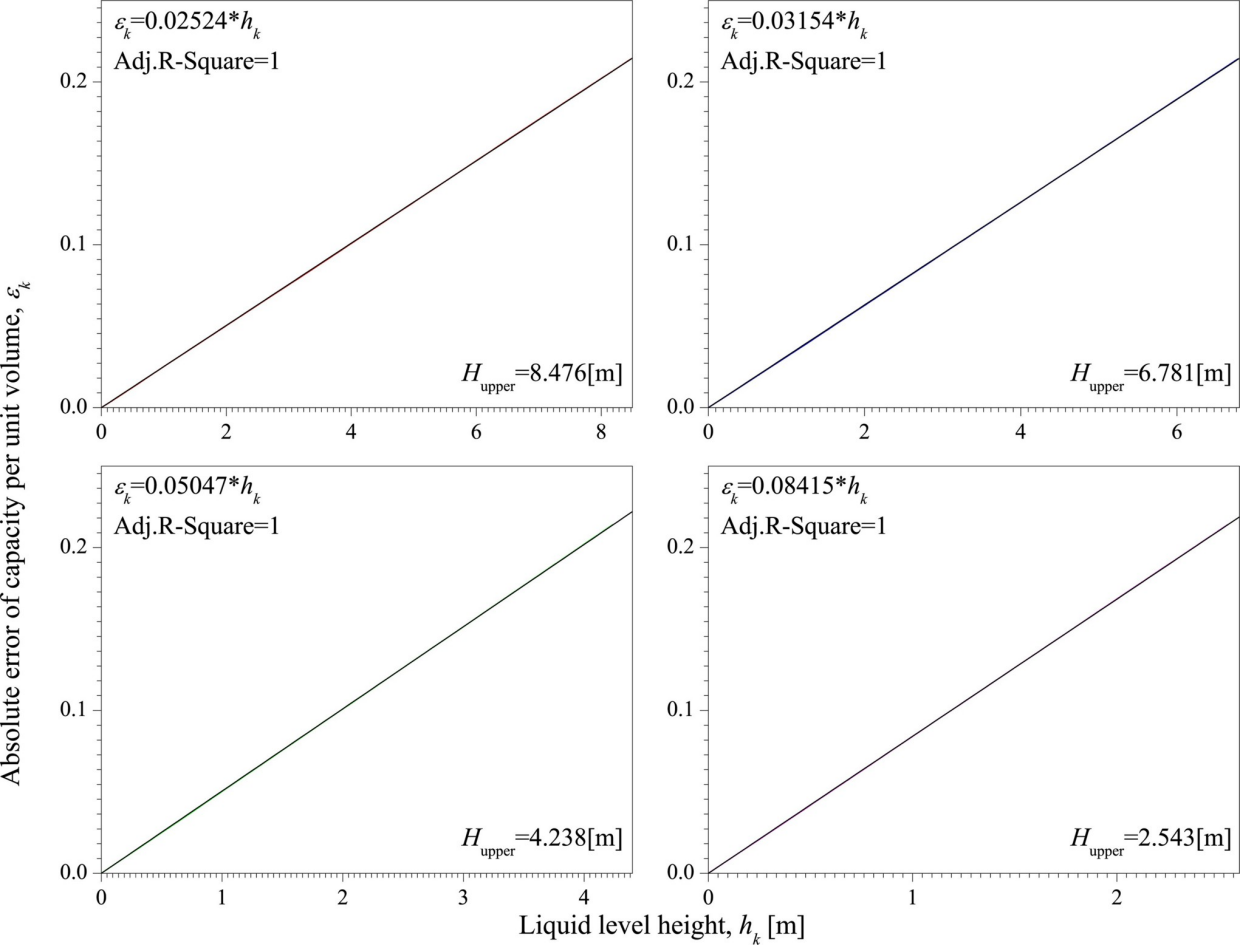
The absolute error of capacity per unit volume with different heights of vertical tank.

**Fig 9** is the absolute error of capacity per unit volume with different heights of vertical tank. The heights of vertical tank are 8.476 [m], 6.781 [m], 4.238 [m], and 2.543 [m]. Each height is tested 10 times and the inner radius of vertical tank is 1.300 [m] in each test. Fig 8 shows the average value of *ε*_*k*_. From the fitting results, even though *H*_upper_ has changed, there is still a significant linear relationship between *ε*_*k*_ and *h*_*k*_. The slopes of the fitting results are different. Interestingly, we find that 0.02524 × 8.476 ≈ 0.214, 0.03154 × 6.781 ≈ 0.214, 0.05047 × 4.238 ≈ 0.214, and 0.08415 × 2.543 ≈ 0.214. This shows that the product of the slope and *H*_upper_ is a constant value, which 0.214.

**Fig 10:**
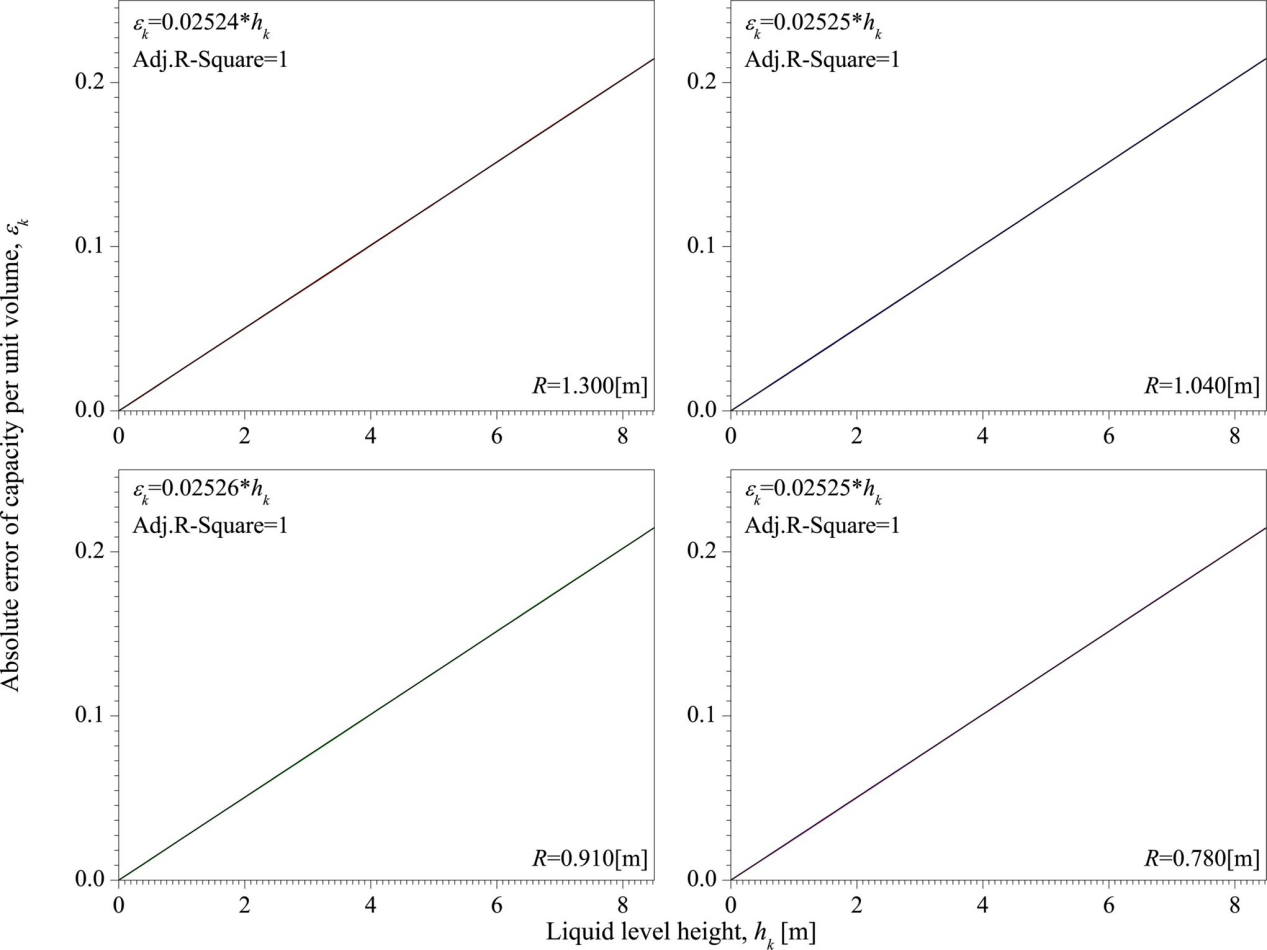
The absolute error of capacity per unit volume with different radii of vertical tank.

**Fig 10** is the absolute error of capacity per unit volume with different radii of vertical tank. The radii of vertical tank are 1.300 [m], 1.040 [m], 0.910 [m], and 0.780 [m]. Each radius is tested 10 times and the height of vertical tank is 8.476 [m] in each test. Fig 10 shows the average value of *ε*_*k*_. It can be seen from Fig 10 that there is a significant linear relationship between *ε*_*k*_ and *h*_*k*_, and the slope hardly changes due to changes in radius.

Based on the above analysis of the influence of *H*_upper_ and *R* on *ε*_*k*_, in the linear relationship between *ε*_*k*_ and *h*_*k*_, *R* has little effect on the slope, and the product of *H*_upper_ and the slope is a constant, which is 0.214. Therefore, the relationship between *ε*_*k*_ and *h*_*k*_ can be written as

$$\varepsilon_{k}=\frac{0.214}{H_{\text{upper}}}\cdot h_{k},$$ (16)

The vertical tank capacity in upper part at *h*_*k*_, which is measured with the Monte Carlo method, can be compensated according to Eq 16. The compensated vertical tank capacity in upper part at *h*_*k*_ is

$${\overset{⌢}{Q}}_{\text{upper-}k}=Q_{\text{upper-}k}-\varepsilon_{k}\cdot L\cdot W\cdot H_{\text{upper}},$$ (17)

Substituting Eq 16 into Eq 17, we have

$${\overset{⌢}{Q}}_{\text{upper-}k}=Q_{\text{upper-}k}-0.214\cdot L\cdot W\cdot h_{k},$$ (18)

Thus,

$${\overset{⌢}{Q}}_{k}=Q_{\text{bottom}}+Q_{\text{upper-}k}-0.214\cdot L\cdot W\cdot h_{k},$$ (19)

### Vertical tank test and result analysis

**Fig 11:**
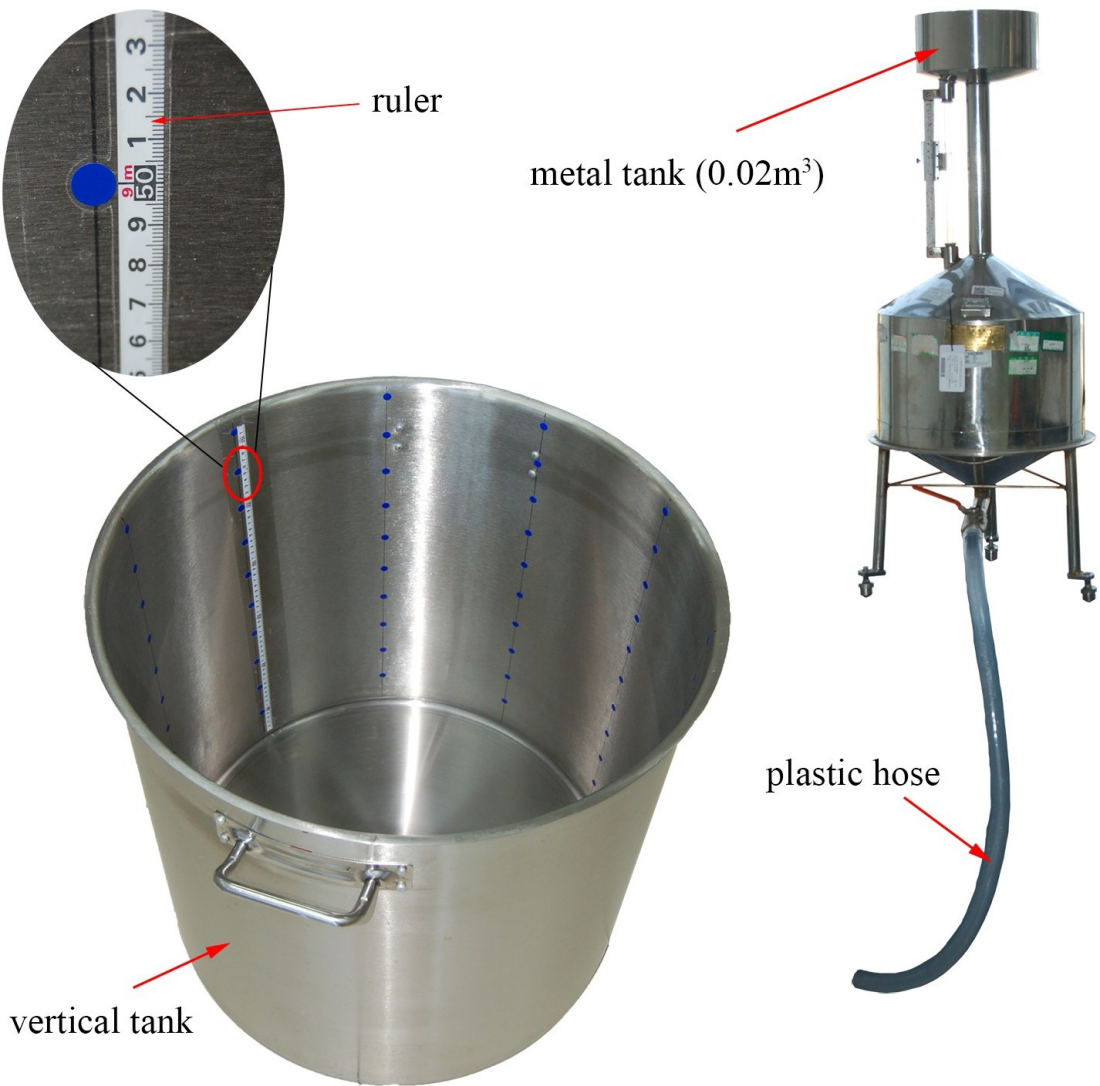
Vertical tank test devices.

**Fig 11** are devices used for vertical tank testing, including a vertical tank [13] and a metal tank [14]. The inner diameter of the vertical tank is *R* = 0.299[m]. One hundred sensor point labels are evenly affixed on the inner wall of the vertical tank, a total of 10 layers, each with 10 points. The distance between the lowest sensor point and the highest sensor point is *H*_upper_ = 0.513[m]. The distance between the sensor points of each layer is *h*_layer_ = 0.057[m]. The capacity of bottom part is *Q*_bottom_ = 0.0175[m^3^]. A ruler is attached to the inner wall of the vertical tank for reading the liquid level with a resolution of 0.001[m]. The nominal capacity of the metal tank is 0.02[m^3^], indicating that it can accurately hold 0.02[m^3^] of liquid. The liquid in the metal tank can be transferred to the vertical tank through the plastic hose. The liquid used in the test is water. Since the nature of water is relatively stable, compared to other media, such as oil, the volume of water is less affected by temperature and volatility, so we used water to verify the accuracy of the proposed method to measure the capacity of vertical tanks.

**Fig 12:**
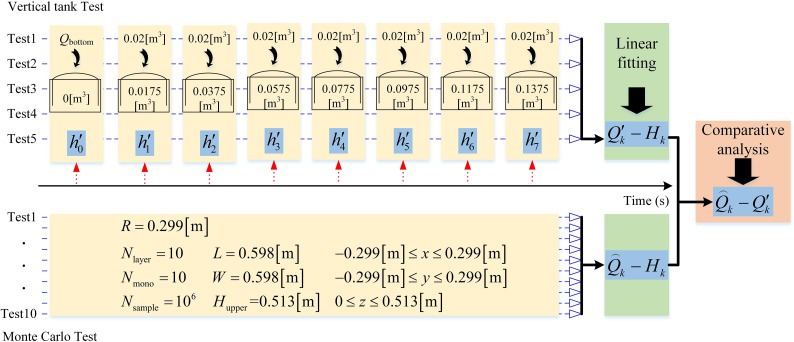
Vertical tank test sequence.

**Fig 12** is the sequence of vertical tank test, including two parts: vertical tank test and Monte Carlo test. In the vertical tank test, the first step is to pour *Q*_bottom_ = 0.0175[m^3^] of water into the tank, and record the liquid level as ${h^{\prime }}_{0}$ at this time. Then 0.02[m^3^] of water is poured into the tank one by one, and the corresponding liquid levels are recorded. The recorded liquid levels are ${h^{\prime }}_{1}$, ${h^{\prime }}_{2}$, ${h^{\prime }}_{3}$, ${h^{\prime }}_{4}$, ${h^{\prime }}_{5}$, ${h^{\prime }}_{6}$, and ${h^{\prime }}_{7}$ in sequence. A total of 5 tests are carried out. In order to prevent the difference of *Q*_bottom_ from affecting the liquid level of the upper part of vertical tank, the liquid levels when the water volume in the vertical tank is 0.0375[m^3^], 0.0575[m^3^], 0.0775[m^3^], 0.0975[m^3^], 0.1174[m^3^], 0.1375[m^3^] and 0.1575[m^3^] are ${h^{\prime }}_{1}-{h^{\prime }}_{0}$, ${h^{\prime }}_{2}-{h^{\prime }}_{0}$, ${h^{\prime }}_{3}-{h^{\prime }}_{0}$, ${h^{\prime }}_{4}-{h^{\prime }}_{0}$, ${h^{\prime }}_{5}-{h^{\prime }}_{0}$, ${h^{\prime }}_{6}-{h^{\prime }}_{0}$ and ${h^{\prime }}_{7}-{h^{\prime }}_{0}$ respectively. Finally, the relationship between the capacity and liquid level of the vertical tank, which is ${Q^{\prime }}_{k}-h_{k}$, is obtained by linear fitting [15]. The ${Q^{\prime }}_{k}-h_{k}$ obtained from the vertical tank test is used as the exact value to verify the effectiveness of the method proposed in this paper.

In the Monte Carlo test, a total of 10 tests are performed, and the parameters of each test are shown in Fig 12. Based on the average value of 10 tests, ${\overset{⌢}{Q}}_{k}-h_{k}$ is calculated using Eqs 13 and 19.

**Fig 13:**
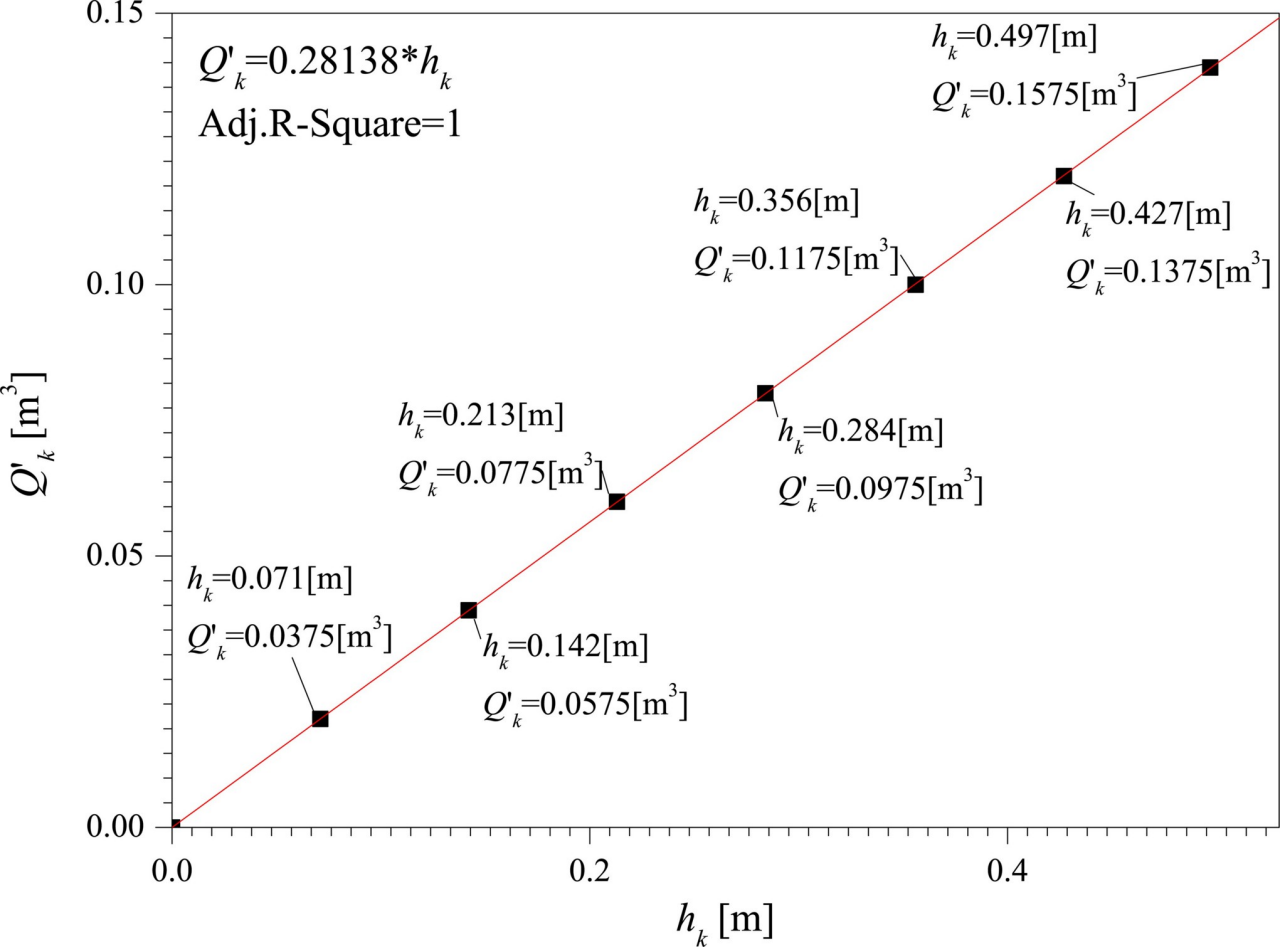
The relationship between the capacity and liquid level of the vertical tank.

Table 1 is the results of vertical tank tests. Based on the average values, the relationship between the capacity and liquid level of the vertical tank is obtained through linear fitting, shown in **Fig 13**.

**Table 1:** Results of vertical tank tests.

	${h^{\prime }}_{1}-{h^{\prime }}_{0}$ [m]	${h^{\prime }}_{2}-{h^{\prime }}_{0}$ [m]	${h^{\prime }}_{3}-{h^{\prime }}_{0}$ [m]	${h^{\prime }}_{4}-{h^{\prime }}_{0}$ [m]	${h^{\prime }}_{5}-{h^{\prime }}_{0}$ [m]	${h^{\prime }}_{6}-{h^{\prime }}_{0}$ [m]	${h^{\prime }}_{7}-{h^{\prime }}_{0}$ [m]
**Test1**	0.072	0.142	0.213	0.285	0.356	0.427	0.498
**Test2**	0.071	0.142	0.213	0.284	0.355	0.426	0.497
**Test3**	0.071	0.142	0.213	0.284	0.356	0.426	0.497
**Test4**	0.071	0.142	0.213	0.284	0.355	0.426	0.497
**Test5**	0.071	0.143	0.214	0.285	0.356	0.427	0.497
**Average**	0.071	0.142	0.213	0.284	0.356	0.427	0.497

**Fig 14:**
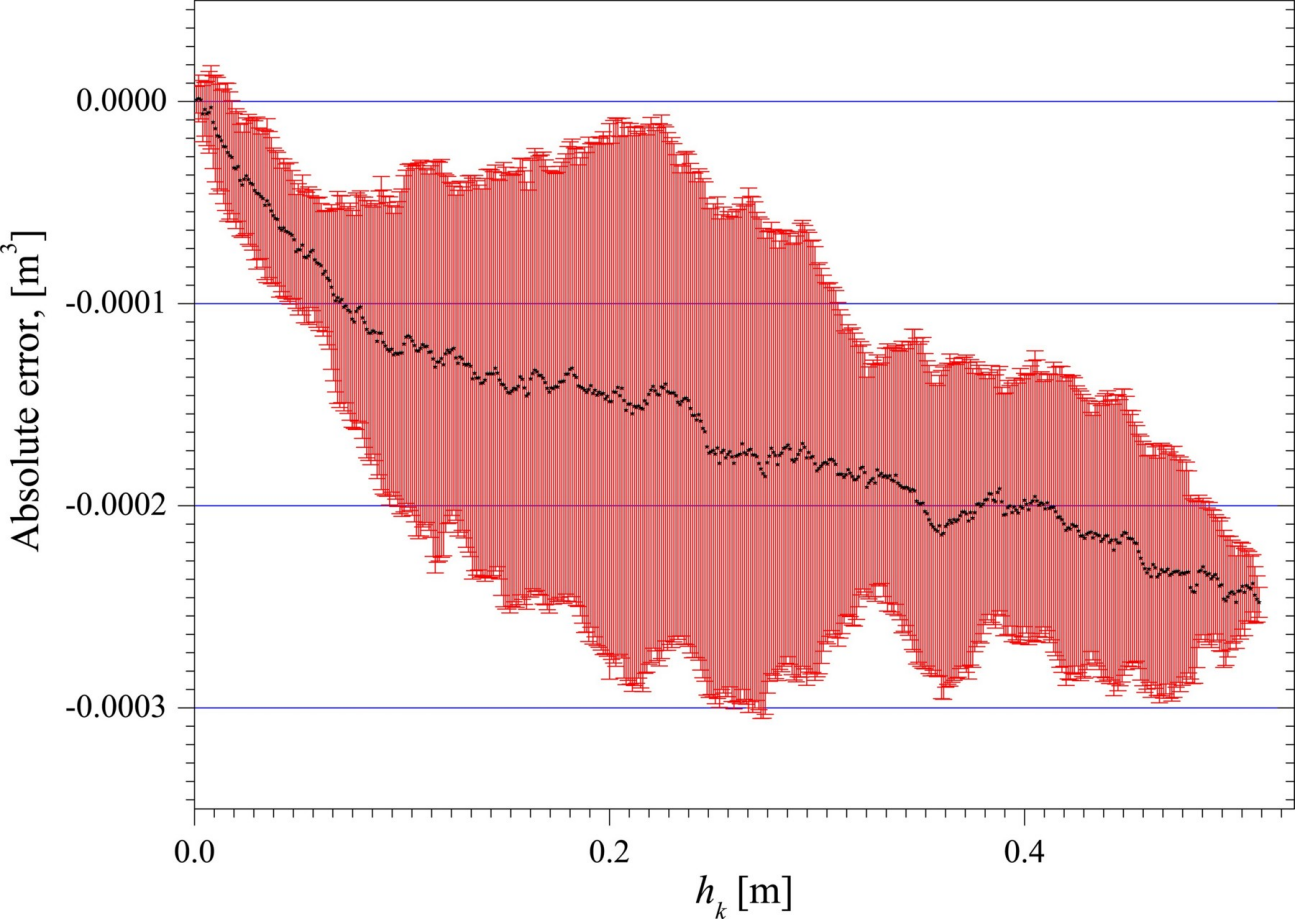
Absolute error distribution.

**Fig 14** is the absolute error distribution of the measurement results obtained through the Monte Carlo method, e.g. ${\overset{⌢}{Q}}_{k}-{Q^{\prime }}_{k}$. In Fig 14, the black mark is the average value of the absolute error of the 10 tests. The red area represents the standard deviation of the absolute error of the 10 tests. The maximum absolute error of using Monte Carlo method to measure the vertical tank capacity does not exceed ±0.0003[m^3^].

**Fig 15:**
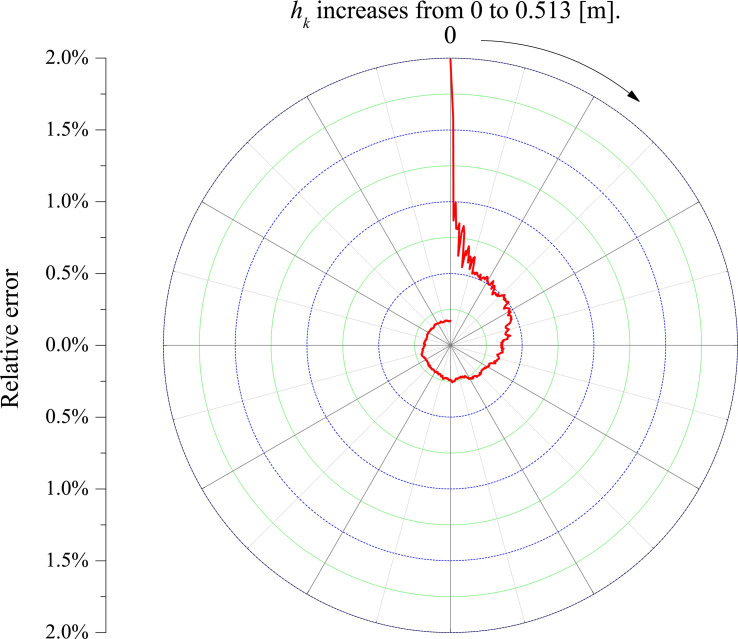
Relative error shrinkage curve.

**Fig 15** is the relative error shrinkage curve, e.g. $\left|{\overset{⌢}{Q}}_{k}-{Q^{\prime }}_{k}\right|/{Q^{\prime }}_{k}$. The relative error finally converges to less than 0.18%.

## Conclusion

A new vertical tank capacity measurement method based on Monte Carlo Method is proposed. Focusing on constructing vertical tank boundary, the method arranges a plurality of sensor points on the inner surface of the vertical tank, and performs Monte Carlo tests by generating a large number of random sample points. The criterions for whether a sample point is located in the vertical tank are established with the coordinates of sensor points and the distance between different sensor points along the surface of the tank. The results show that the absolute error of capacity per unit volume has a linear relationship with the height of the vertical tank, and has little effect with the radial size of the vertical tank. The absolute error of the measurement results of the proposed method does not exceed ±0.0003[m^3^], and the relative error finally converges to less than 0.18%.

Although the method we proposed can effectively measure the capacity of vertical tank, there are still some limitations that need to be improved. The number of sensor points is large, and the arrangement of the sensor points is relatively neat. We will further explore the influence of the number and location of sensor points on the measurement results in the future, try to reduce the number of sensor points, and more convenient ways to arrange the sensor points, such as scattered random arrangement. In addition, this method is suitable for tanks of various shapes, such as cuboid, cylinder, etc., but the connection between any two sensor points must be inside the tank, and there can be no such connection outside the tank. At this stage, this article mainly discusses the feasibility of the proposed method. In the future application stage, there are still many problems to be solved, such as the selection of sensors and the reaction of sensors with liquids. Regarding the selection of sensors, we have made some guesses. Surface acoustic wave sensors, ultrasonic guided wave sensors, and stress-strain sensors may be suitable.

## Supporting information

S1 File Dataset. (XLSX)
